# Efficacy of Low-Dose rhGM-CSF Treatment in a Patient With Severe Congenital Neutropenia Due to CSF3R Deficiency: Case Report of a Novel Biallelic CSF3R Mutation and Literature Review

**DOI:** 10.3389/fped.2021.746159

**Published:** 2021-10-29

**Authors:** Junli Zhou, Chengjun Sun, Honglin Huang, Qiguo Zhu, Fengyun Wen, Ying Dong, Hongsheng Wang

**Affiliations:** ^1^Departments of Cardiology, Endocrine, Hematology and Nephrology, Children's Hospital of Fudan University at Xiamen, Xiamen Children's Hospital, Xiamen, China; ^2^Department of Endocrinology and Inherited Metabolic Diseases, National Children's Medical Center Children's Hospital of Fudan University, Shanghai, China; ^3^Department of Hematology, National Children's Medical Center Children's Hospital of Fudan University, Shanghai, China

**Keywords:** severe congenital neutropenia, biallelic heterozygous, *CSF3R*, rhGM-CSF, treatment

## Abstract

This study reports the clinical manifestations, genetics, and efficacy of treatment with the efficacy of recombinant human granulocyte macrophage colony-stimulating factor (rhGM-GSF) of a 2-year-old female patient with severe congenital neutropenia (SCN) type 7 (SCN7) caused by novel biallelic mutations in the colony-stimulating factor 3 receptor (*CSF3R*) gene. Genetic diagnosis of the patient was performed by whole-exome and Sanger sequencing. Expression of the *CSF3R* gene in the peripheral neutrophils of the patient was detected by real-time PCR and Western blotting. The patient presented with recurrent suppurative tonsillitis and decreased absolute neutrophil count <0.5 × 10^9^/L. Novel heterozygous mutations were found to be inherited from each parent (maternal c.690delC [p.met231Cysfs^*^32] and paternal c.64+5G>A). The patient's neutrophils had lower *CSF3R* mRNA and protein levels than those of the parents. Low-dose rhGM-CSF (3 μg/kg/day once a week) prevented recurrent infection in the patient. These results demonstrate that the clinical manifestations of SCN7 with biallelic *CSF3R* mutations and downregulated *CSF3R* can be effectively treated with rhGM-CSF.

## Introduction

Severe congenital neutropenia (SCN) is a rare hematologic disease characterized by severely reduced numbers of the peripheral blood neutrophils (absolute neutrophil count [ANC] <0.5 × 10^9^/L) along with recurrent bacterial infection and increased risk of malignancies (1, 2). The major types of SCN (SCN types 1–8) are caused by mutations in the following genes: *ELANE, GFI1, HAX1, G6PC3, VPS45, JAGN1, CSF3R*, or *SRP54* (1, 3). Most patients with SCN can be effectively managed with injections of granulocyte colony-stimulating factor (G-CSF) injections, which stimulate cell proliferation and differentiation into mature neutrophilic granulocytes (4–6). However, a small proportion of SCN patients with G-CSF receptor deficiency (i.e., due to biallelic loss-of-function mutations in the *CSF3R* gene) are refractory to G-CSF but respond to granulocyte macrophage colony-stimulating factor (GM-CSF) treatment (7, 8). Here, we describe a case of SCN arising from novel biallelic heterozygous *CSF3R* mutations and review other cases reported in the literature.

## Materials and Methods

### Case Description

A 2-year-old girl initially presented at the hospital during an episode of recurrent suppurative tonsillitis (9). Physical examination revealed moderately enlarged tonsils but no signs of systemic or severe infection. Her complete blood count (CBC) showed a marked decrease in ANC (0.42 × 10^9^/L [normal range: 2.4–4.8 × 10^9^/L]) and mild anemia (white blood cell [WBC] 4.0 × 10^9^/L [normal range: 6–12 × 10^9^/L]; hemoglobin (Hb) 94 g/L [normal range: 110–160 g/L]; and platelet [PLT] 257 × 10^9^/L [normal range: 100–400 × 10^9^/L]). She had an increased C-reactive protein (CRP) level (170 mg/L [normal range:0–10 mg/L]), procalcitonin (0.14 ng/ml [normal range: 0–0.06 ng/ml]), and erythrocyte sedimentation rate (83 mm/h [normal range: 0–15 mm/h]). Further laboratory tests showed that her serum antistreptolysin O level was normal, and no Epstein–Barr virus DNA was detected in her peripheral blood. Her serum immunoglobulin (IgG, IgM, and IgA) levels were normal. Flow cytometry analysis showed she had a normal peripheral lymphocyte profile (cluster of differentiation 3-positive [CD3^+^], CD19^+^, CD56^+^, CD3^+^CD4^+^, and CD3^+^CD8^+^ cell counts and CD4^+^/CD8^+^ ratio were all in normal ranges for healthy children).

The patient recovered after 1 week of treatment with antibiotics. CBC after her recovery confirmed neutropenia (WBC 5.6 × 10^9^/L, Hb 104 g/L, PLT 349 × 10^9^/L, ANC 0.19 × 10^9^/L, CRP 9.95 mg/L). Cytologic analysis of bone marrow aspirate showed normal cellularity in terms of the proportion of cells in each granulocyte stage and cell morphology. A review of her medical history confirmed that she had low ANC (0.4 × 10^9^/L) starting from 5 months of age but was refractory to recombinant human (rh)G-CSF treatment (15–30 episodes of suppurative tonsillitis between the ages of 1 and 2 years under rhG-CSF treatment). Her parents denied consanguinity and both were healthy with normal ANC.

**Figure 1:**
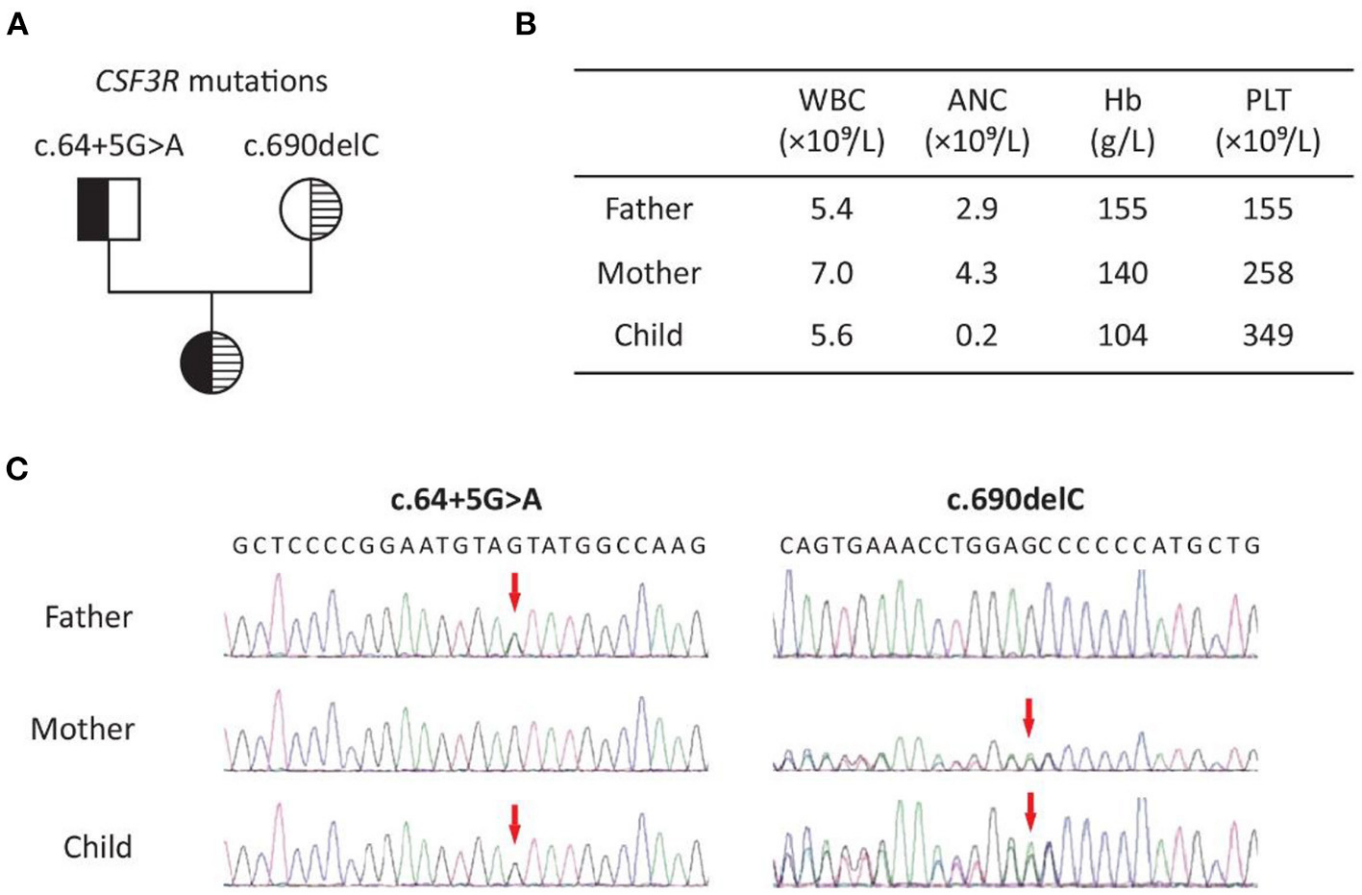
Patient with SCN7 due to biallelic *CSF3R* mutations: **(A)** pedigree; **(B)** representative results of complete blood count in the family; **(C)** Sanger sequencing chromatogram of c.64+5G>A and c.690delC (NM_000760.3). WBC, white blood cell; ANC, absolute neutrophil count; Hb, hemoglobin; PLT, platelet.

Whole-exome sequencing of the patient and her parents identified a compound heterozygous mutation in the *CSF3R* gene (NM_000760.3) in the child, who inherited an intronic mutation (c.64+5G>A) from her father and a frameshift mutation (c.690delC, p.Met231Cysfs^*^32) from her mother (Figure 1). The latter was predicted to be damaging by MutationTaster (http://www.mutationtaster.org/). The clinical significance of the intronic mutation (c.64+5G>A) is not clear. Both mutations are present at a very low frequency in the general population (Table 1).

**Table 1:** Mutations in *CSF3R* (NM_000760.3) identified in the patients with congenital severe neutropenia type 7.

**Genetic mutation**	**Position (GRCh37/38)**	**GnomeAD population frequency**	**Protein change**	**Child**	**Father**	**Mother**	**ACMG category**
c.690delC	chr1:36938271/36472670	0	p.Met231Cysfs*32	del/C	C/C	del/C	PVS1
c.64+5G > A	chr1:36945029/36479428	0	?	G/A	G/A	G/G	PM2

The expression level of *CSF3R* in neutrophils of the patient and her parents was determined by real-time (rt)PCR and Western blotting. In rtPCR, total RNA extracted from peripherial blood using TRIzol reagent (Invitrogen, Carlsbad, CA, USA) was reverse transcribed to cDNA using the PrimeScript RT reagent kit (Takara Bio, Ostu, Japan). The rtPCR was performed on a LightCycler 480 system (Roche, Basel, Switzerland) using the SYBR Premix ExTaq Kit (Takara Bio). The 2^−ΔΔCt^ method was used to calculate the relative levels of *CSF3R* mRNA. Primer sequences (Sangong Biotech, Shanghai, China) are 5′-GGGGA GAGAA GCTGG ACTG-3′ and 5′-GTTCA TGAGG CAGGA GAGGT-3′ for *CSF3R* (product length 578 bp), and 5′-ACATC AAGAA GGTGG TGAAG-3′, and 5′-TGACA AAGTG GTCGT TGAG-3′ for *GAPDH* (product length 220 bp). For Western blotting, total protein was extracted from neutrophils separated from peripherial blood of the of the patient and her parents using a commercial kit (protein extraction kit [BB-3134], BestBio, Shanghai, China). After determining the protein concentration with the BCA method, whole protein extract was resolved by SDS-PAGE using a 4% acrylamide gel. The proteins were transferred to a PVDF membrane (Millipore, Schwalbach, Germany) that was probed with primary antibodies against CSF3R (ab156878) and GAPDH (ab8245) followed by horseradish peroxidase-conjugated goat antirabbit IgG H&L (ab205718, all from Abcam, Cambridg, UK). Protein bands were visualized by enhanced chemiluminescence using an imaging system (Tanon 6600, Science & Technology Co, Shanghai, China). Quantification of band intensities in Western blotting was performed using ImageJ (version 1.53k) (10). The levels of CSF3R expression in neutrophils between subjects were compared by *t-*test (R, version 4.0.3, www.r-project.org), and a *p* < 0.05 is considered statistically significant.

## Results

### Diagnosis and Treatment

**Figure 2:**
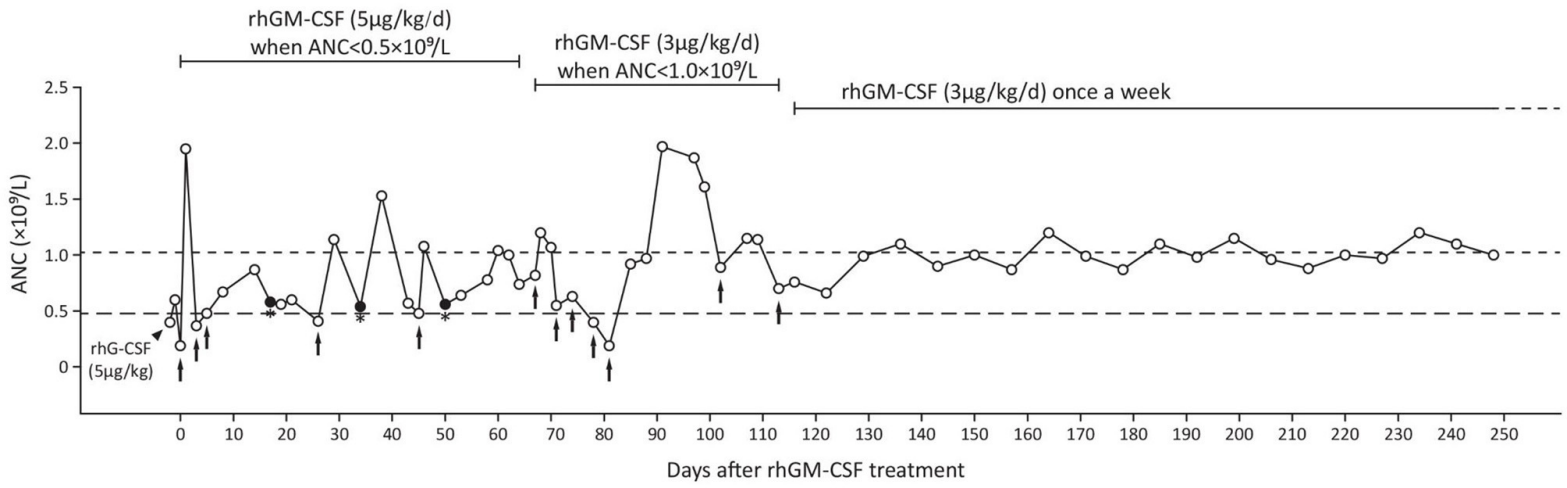
The patient's treatment using rhG-CSF (black triangle) and rhGM-CSF (black arrows indicate treatment initiation and injections before regular treatment). Closed dot with asterisk suggests infection. ANC, absolute neutrophil count; rhG-CSF, recombinant human granulocyte colony-stimulating factor; rhGM-CSF, recombinant human granulocyte macrophage colony-stimulating factor.

Based on the patient's presentation and the family's genetic profile, SCN type 7 (SCN7, MIM #617014) was diagnosed. After initiating rhG-CSF (5 μg/kg) treatment, the patient's ANC increased from 0.4 to 0.6 × 10^9^/L; however, after rhGM-CSF (5 μg/kg, Topleucon, Amoytop Biotech, Xiamen, China) injection, ANC increased from 0.19 to 1.95 × 10^9^/L (Figure 2). Therefore, the treatment plan was changed to subcutaneous rhGM-CSF (5 μg/kg/day) once ANC was lower than 0.5 × 10^9^/L (on twice a week surveillance). During the next 68 days, the patient received five rhGM-CSF subcutaneous injections but three episodes of suppurative tonsillitis occurred. After observing that overt infection still occurred when ANC was >0.5 × 10^9^/L, we raised the ANC target to 1.0 × 10^9^/L with a decreased dose (3 μg/kg/day) and increased frequency (twice a week) of rhGM-CSF. The patient received eight injections during the next 42 days, and no infection occurred. We then adjusted the injection interval from twice to once a week and monitored ANC once a week before injection and continued to follow up the patient for the next 6 months. There were no other occurrences of infection (Figure 2).

### The Expression of *CSF3R* in Peripherial Neutrophils of the Patient

**Figure 3:**
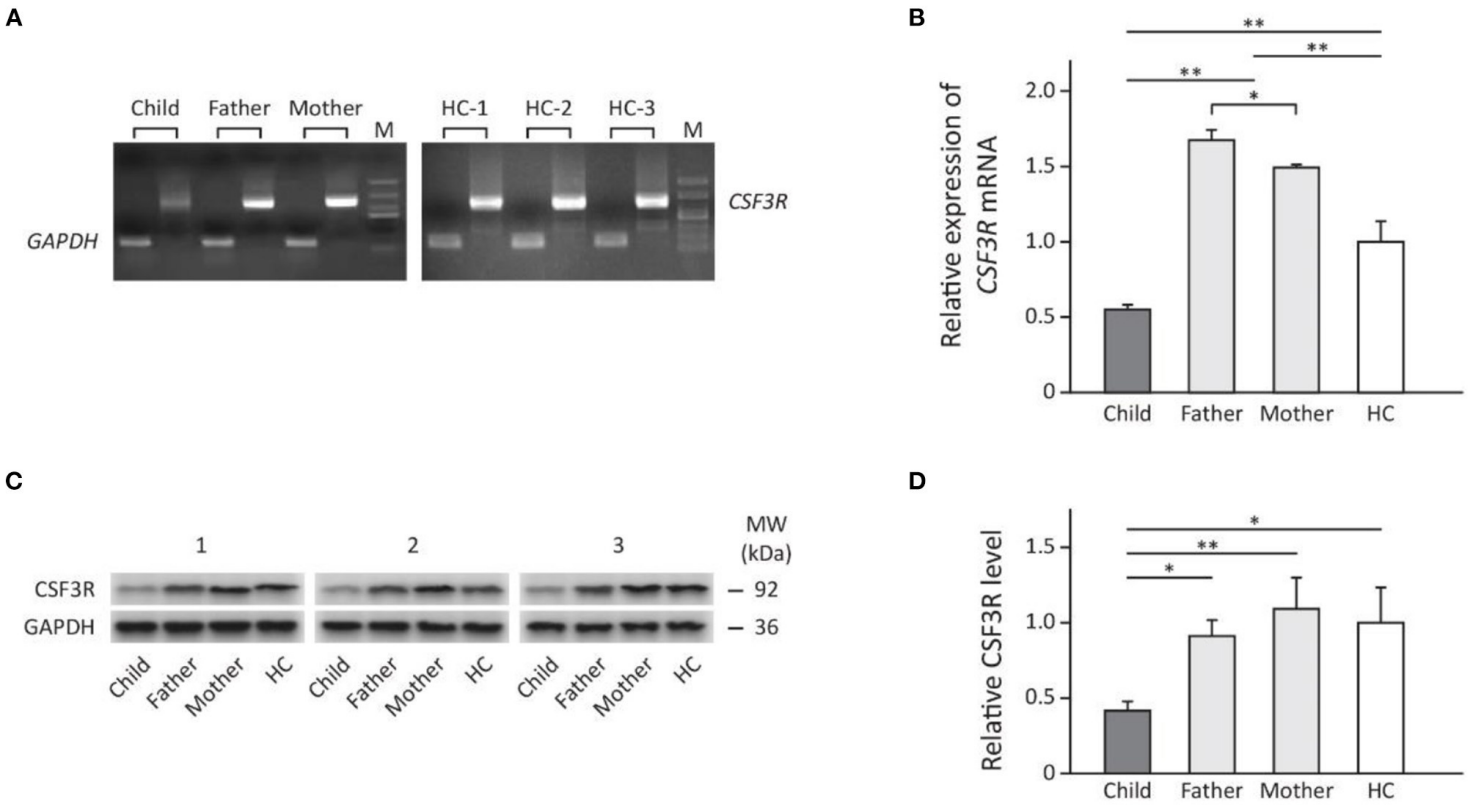
The expression of *CSF3R* in the family measured by: **(A)** direct PCR using cDNA transcription of total RNA extracted from subject's neutrophils; **(B)** rt-PCR showing fold change of relative *CSF3R* expression (normalized to *GAPDH*) compared to healthy controls. **(C)** Western blot (*n* = 3) and **(D)** the relative levels of CSF3R protein in neutrophils adjusted by GAPDH (Quantification of band intensities was performed using ImageJ). Results are presented as mean ± SD, *p < 0.05, **p < 0.01. HC, healthy control; M, marker; MW, molecular weight.

The mRNA and protein levels of *CSF3R* in perpherial neutrophils were significantly lower in the patient when compared to either her parents or healthy controls (Figure 3). However, parents of the patient showed significantly increased *CSF3R* mRNA than control subjects but the CSF3R protein level was similar to that of healthy controls (Figure 3).

### Literature Review

We searched the literature for the clinical characteristics and treatment of all cases with SCN7 caused by biallelic *CSF3R* mutation reported since 2014 (Table 2). There are nine pedigrees (13 patients) that have been described to date; six cases were from Turkey (11), nine were female, and all presented repeated infection and required long-term antibiotic treatments. One patient died shortly after birth for unknown reasons (12). The phenotype of SCN7 ranged from severe neutropenia unresponsive to high-dose rhG-CSF treatment (c.998-2A>T and p.W547^*^ compound heterozygous) (7) to mild neutropenia that does not require active treatment (p.R440^*^ homozygous) (14). Among the three patients (including patient in current study) who responded to rhGM-CSF treatment, the dose of rhGM-CSF ranged from 3 μg/kg every week to 5 μg/kg every other day (7, 8).

**Table 2:** The clinical characteristics and treatment of children with congenital neutropenia caused by biallelic *CSF3R* mutations in the literature.

**No**.	**Ethnicity**	**Sex**	**Age at onset**	**Age at diagnosis**	**ANC (**×10^9^L**) before treatment**	**Bone marrow aspiration**	**rhG-CSF**	**rhGM-CSF**	**Genetics**	**Outcome**	**Reference**
1	Turkish	M	NA	2.5 Y	NA	Normal	5 μg/kg/d (unresponsive)	NA	c.922C>T (p.Arg308Cys) hom	NA	
2	Turkish	F	At birth	At birth	0.42–2.18	Normal	5 μg/kg/d (unresponsive)	NA	c.922C>T (p.Arg308Cys) hom	NA	
3	Turkish	F	At birth	At birth	NA	NA	NA	NA	c.922C>T (p.Arg308Cys) hom	Died at age 3 months	Yilmaz Karapinar et al. (11)
4	Spanish	F	2 M	9 M	0.2–1.0	Normal	40 μg/kg/d (unresponsive)	NA	c.948_963del (p.Gly316fsTer322) c.1245del (p.Gly415fsTer432)	NA	Triot et al. (12)
5	NA	F	At birth	At birth	<0.25	Normal	110 μg/kg/d (unresponsive)	6–3 μg/kg/d biw	c.998-2A>T (p.W547*)	Regular rhGM-CSF for 12 years	Klimiankou et al. (7)
6	NA	F	4 M	3.5 Y	0.041–0.508	Normal	15 μg/kg/d (unresponsive)	5 μg/kg/d qod	c610-611 del ins AG (p.Q204R) hom	Regular rhGM-CSF for 4 months	Yilmaz Karapinar et al. (8), Yilmaz Karapinar et al. (11)
7	Turkish	F	9 M	4 Y 3 M	0.167–1.1	Normal	5 μg/kg/d (responsive)	NA	c.266T>C (p.L89P) c.569G>A (p.R190H)	Treatment on demand	Yilmaz Karapina et al. (13), Yilmaz Karapinar et al. (11)
8	Tukish	NA	NA	NA	<0.55	NA	NA (unresponsive)	NA	c.1765G>A(p.G589S)hom	NA	Yilmaz Karapinar et al. (11)
9	Tukish	NA	NA	NA	<0.55	NA	NA (unresponsive)	NA	c.1765G>A(p.G589S)hom	NA	
10	Cyprus / England	F	Childhood	40 Y	0.5–2.0	reduced granulocyte progenitors	NA (unresponsive)	NA	c.359G>A(p.G120D)c.1640G>A(p.W547)	NA	
11	Turkish	M	Childhood	5 Y	0.5–1.5	NA	NA	NA	c.1318C>T(p.R440*)hom	Untreated	Sprenkeler et al. (14)
12	Turkish	F	Childhood	11 Y	0.7–1.6	NA	NA	NA	c.1318C>T(p.R440*)hom	Untreated	
13	Chinese	F	5 M	2 Y	0.2–0.5	Normal	5 μg/kg/d (unresponsive)	3 μg/kg/d qw	c.690delC c.64+5G>A	Regular rhGM-CSF for 6 months	Current

*No., number; M, male; F, female; NA, not available; Y, year; M, months; ANC, absolute neutrophils count; rhG-CSF, recombinant human granulocyte colony-stimulating factor; rhGM-CSF, recombinant human granulocyte-macrophage colony-stimulating factor; biw, twice a week; qod, every other day; qw, once a week; hom, homozygous*.

## Discussion

In this report, we describe the case of a 2-year old girl with recurrent infection and neutropenia who was genetically diagnosed with SCN7 caused by biallelic inactivating mutations in *CSF3R*. Similar to most previously reported cases, our patient did not respond to rhG-CSF, but rhGM-CSF treatment showed satisfactory efficacy and was titrated to a lower dose (3 μg/kg/day once a week).

SCN is a group of rare congenital hematologic diseases caused by germline mutations with an incidence rate of ~1/200,000 (15). To date, more than 20 SCN-related genes have been identified, including *ELANE* (16), *GFI-1* (17), *HAX1* (18), *G6PC3* (19), *VPS45* (20), *JAGN1* (21), and *CSF3R* (7, 22). The mortality of SCN is attributable to an increased risk of severe bacterial infection (23). Since 1987, the use of rhG-CSF has significantly improved the prognosis and quality of life of patients with SCN (24, 25). In most patients, the frequency of bacterial infection is significantly reduced when ANC is maintained above 1.0 × 10^9^/L (26).

G-CSF and its receptor are required to maintain normal neutrophil numbers during basal and emergency granulopoiesis in humans, mice, and zebrafish (27–29). The ineffectiveness of G-CSF in patients with SCN7, which is caused by biallelic loss-of-function in the G-CSF receptor gene (*CSF3R*) at a high dose (up to 110 μg/kg/day) (7), demonstrates the importance of interaction between G-CSF and its receptor for the maintenance of normal neutrophil numbers.

CSF3R dimerizes to form a functional receptor (7). A previous report shows that patients with bialliac CSF3R deficiency can present mild neutropenia that does not require active treatment (14) and suggests that other compensatory mechanisms are involved in the maintenance of adequate number of neutrophils (30). Our results show that *CSF3R* mRNA expression was decreased in the patient but was increased in both parents who were heterozygous carriers; on the other hand, Western blot analysis showed that the parents had normal CSF3R protein levels. This is similar to previous findings that the expression of nonfunctional CSF3R was increased in patients with biallelic *CSF3R* mutations (12), and suggests that a genetic compensation mechanism rather than non-sense-mediated decay was activated to maintain the normal CSF3R levels (31, 32).

Both G-CSF and GM-CSF play important roles in the differentiation and development of myeloid cells. The responsiveness to rhGM-CSF of patients with *CSF3R* mutations can be attributed to granulocyte stimulation by GM-CSF and the activation of CSF2R (30, 33, 34). GM-CSF sequentially induces myeloid proliferation, differentiation, and maturation (35) with mature neutrophils released into the peripheral circulation after about 5–8 days (36). We believe that rhGM-CSF (3 μg/kg/day) once a week can be effective for maintaince when the patient is free from overt infection (i.e., no additional neutrophil consumption). However, the current cases are not sufficient to summarize a clear relation between genotype and responsiveness to rhGM-CSF, an individualized treatment plan based on the severity of infection, mutation type, and response to treatment is probably needed for each patient. The current literature did not confirm whether a patient with SCN7 can be unresponsive to either rhG-CSF or rhGM-CSF, and among those patients, hematopoietic stem cell transplantation can serve as a treatment option (37, 38).

## Conclusions

In summary, our study shows that patients with SCN7 caused by biallellic *CSF3R* mutations with decreased CSF3R expression respond well to adequate rhGM-CSF treatment. Low-dose GM-CSF once weekly is capable of maintaining our patient free from recurrent infection.

## Data Availability Statement

The original contributions presented in the study are included in the article/supplementary materials, further inquiries can be directed to the corresponding author.

## Ethics Statement

The studies involving human participants were reviewed and approved by Ethics Committee of Children's Hospital of Fudan University. Written informed consent to participate in this study was provided by the participants' legal guardian/next of kin. Written informed consent was obtained from the minor(s)' legal guardian/next of kin for the publication of any potentially identifiable images or data included in this article.

## Author Contributions

JZ, CS, and HW designed the study and wrote the manuscript. JZ and CS performed literature review. JZ performed the experiments and analyzed the data. HH, QZ, FW, and YD participate in the design of experiments. All authors manuscript formatting and editing.

## Funding

This work was supported by Shanghai Municipal Commission of Health and Family Planning Scientific Research General Program (No. 201640141) and the “1125 talent project” of Xiamen Children's Hospital of China.

## Conflict of Interest

The authors declare that the research was conducted in the absence of any commercial or financial relationships that could be construed as a potential conflict of interest.

## Publisher's Note

All claims expressed in this article are solely those of the authors and do not necessarily represent those of their affiliated organizations, or those of the publisher, the editors and the reviewers. Any product that may be evaluated in this article, or claim that may be made by its manufacturer, is not guaranteed or endorsed by the publisher.
